# A retrospective review of the community medicine needs from osteoporosis services in Canada

**DOI:** 10.1186/s12902-022-01000-y

**Published:** 2022-03-26

**Authors:** Gregory A. Kline, Christopher J. Symonds, Emma O. Billington

**Affiliations:** 1 Dr. David Hanley Osteoporosis Centre, 1820 Richmond Rd SW, Calgary, AB T2T 5C7 Canada; 2 Cumming School of Medicine, University of Calgary, Calgary, Canada

**Keywords:** Osteoporosis, Metabolic bone disorders, Fractures, Bisphosphonates, Medical education, Knowledge translation, Osteoporosis guidelines

## Abstract

**Background:**

Comprehensive, real-world osteoporosis care has many facets not explicitly addressed in practice guidelines. We sought to determine the areas of knowledge and practice needs in osteoporosis medicine for the purpose of developing an osteoporosis curriculum for specialist trainees and knowledge translation tools for primary care.

**Methods:**

This was a retrospective review of referral questions received from primary care and specialists to an academic, multi-disciplinary tertiary osteoporosis and metabolic bone clinic. There were 400 referrals in each of 5 years (2015–2019) selected randomly for review. The primary referral question was elucidated and assigned to one of 16 pre-determined referral topics reflecting questions in the care of osteoporosis and metabolic bone patients. The top 7 referral topics by frequency were determined while recording the referral source.

**Results:**

The majority of referrals (71%) came from urban primary care. The most common specialists to request care included rheumatology, oncology, gastroenterology and orthopedic surgery (fracture liaison services). Primary care referrals predominantly requested assistance with routine osteoporosis assessments, bisphosphonate holidays, bisphosphonate adverse effects/alternatives, fractures occurring despite therapy and adverse changes on bone densitometry despite treatment. Specialists most often referred patients with complex secondary bone diseases or cancer. The main study limitation was that knowledge needs of referring physicians were inferred from the referral question rather than tested directly.

**Conclusion:**

By assessing actual community demand for services, this study identified several such topics that may be useful targets to develop high quality knowledge translation tools and curriculum design in programs training specialists in osteoporosis care.

**Supplementary Information:**

The online version contains supplementary material available at 10.1186/s12902-022-01000-y.

## Introduction

Age-related osteoporosis and fragility fractures are the most common forms of metabolic bone problems in modern medicine [1], affecting 16 to 30% of people over the age of 50 with higher rates in those over 80 years old [2]. To assist primary care providers in clinical practice, many countries have regularly updated osteoporosis practice guidelines to optimize the detection, assessment and treatment of individuals at the highest risk of fracture [3–6]. However, the actual clinical practice of bone medicine is necessarily more complex than may be addressed in general osteoporosis guidelines. Therefore, while many aspects of osteoporosis care have been managed in the primary care setting, bone medicine specialists are called upon to address less-common metabolic bone disorders and to support clinical decisions within areas of controversy related to osteoporosis care.

It is important to understand the needs of a population in order to develop resources required to address the issues of patients in both primary and specialist care. Osteoporosis-related knowledge translation programs directed at primary care audiences may benefit from objective measures that can reliably guide topics for continuing professional development and areas for guideline writers to address. Additionally, residency training programs in internal medicine/geriatrics, endocrinology and rheumatology may benefit from an osteoporosis-specific curriculum that focusses on the measured needs of the community. In this way, both primary and specialist care providers may be prepared to work together for the majority of community needs.

To categorize and quantify the community osteoporosis needs, as defined by the real-world practice, we conducted a detailed, retrospective review of a large sample of referrals sent to a multi-disciplinary metabolic bone and osteoporosis clinic serving both urban and rural primary care as well as other medical sub-specialists. The primary aim was to determine the most common needs and knowledge care gaps in osteoporosis medicine, in order to inform future guideline writers and medical curriculum developers.

## Methods

This study was approved by the ARECCI ethics board of Alberta Innovates; as a retrospective quality-focused study, waiver of individual patient consent was granted and study conduct followed the relevant guidelines and regulations. The Dr. David Hanley Osteoporosis Centre (DHOC) is a multi-disciplinary clinic in Calgary, Canada, devoted to the care of all forms of osteoporosis/metabolic bone disease in Southern Alberta and surrounding area, serving a population of at least 1.3 million people. The clinic is staffed by 5 physicians with expertise in bone medicine, along with a dedicated bone pharmacist, nurse-clinician, dietitian and clinical support staff. The DHOC offers a wide range of in-person and online education for patients and the general public, in addition to individual consultation services. The DHOC receives referrals directly from both primary care and specialist physicians as well as indirectly in transfer from the Central Access and Triage program of the Endocrinology and Rheumatology divisions (Calgary Zone), of Alberta Health Services.

In order to capture the consultative needs of the referring community, we conducted a manual review of referrals received at the DHOC between the years of 2015 to 2019. A sample size of 400 referrals per year was selected in order to capture the frequency of even rare referral questions. Original referrals to the DHOC are maintained in a secure central database categorized by year and in 6-month blocks (January-June, July-December). Individual referral files are kept in alphabetical order of last name within each block. In order to facilitate non-selective sampling, the first 400 alphabetical referrals from the first block of each year were selected for review. Each referral was reviewed in detail by a single reviewer (GK) with long-term experience in osteoporosis medicine and data manually abstracted for analysis. Basic demographic data was collected and after reading the referral text, the reviewer assigned the data to one of 16 pre-defined, possible categories depending on the primary referral question alone; the presence of additional factors that could possibly be congruent with another referral category were not considered unless they specifically factored in the primary question written by the referring physician. For example, “please see this 72 year old woman who just broke her wrist despite being on alendronate for 11 years,” contains data that could be relevant to a question of bisphosphonate holiday but the immediate, primary question being asked is around the issue of a patient sustaining a fracture despite treatment. A referral could be categorized into more than one referral type but only if the referral contained explicit wording of more than one question; simply inferring a second question was not permitted.

Referral question categories were derived from prior pilot data previously gathered during the routine clinical triage process and are outlined, with examples and narrative explanations, in Table 1. The original case categorization was developed informally as a means of assessing the various levels of acuity and complexity in the referrals being received, for the purpose of informing referral triage processes and clinical resource allocation. Data was analyzed using descriptive statistics as both a single dataset and according to annual blocks. ANOVA comparison of multiple continuously distributed non-parametric data was performed by the Kruskal–Wallis test and Dunn’s multiple comparisons test with multiply-adjusted p-value used for comparison of pairs within the ANOVA columns. Where comparison of proportions was performed, a chi-square test was used with the level of statistical significance set at *p* < 0.05. Statistical analysis was performed using GraphPad Prism 6.0 (LaJolla, California) and the project was approved by the ARECCI Quality Improvement program of Alberta Innovates; there was no external funding.

**Table 1:** Referral question categories with narrative examples

REFERRAL QUESTION CATEGORY	NARRATIVE EXAMPLE	DIRECTLY STATED OR INFERRED ISSUE
Recent fracture, no therapy started yet	“70 year old with 2 recent compression fractures” “50 year old man with incidental compression fracture found on x-ray” “53 year old woman with wrist fracture but normal bone density”	Uncertainty about whether fracture is “osteoporotic” Uncertainty about whether treatment indicated Implied that severity needs specialist care Referral from non-prescribing specialist (i.e. orthopedic surgery)
Recent fracture despite therapy	“84 year old with vertebral fracture despite risedronate” “75 year old with 3 metatarsal fractures on denosumab”	Implied that fracture means therapy failure Implied that specialist investigation is needed Implied that fracture defines need to switch treatment drugs Implied that fracture on therapy is abnormal, requires specialist review
Bone disease in context of CKD	“38 year old with type 1 diabetes on dialysis with hip fracture” “77 year old with low BMD and eGFR 27 ml/min/m2”	Recognition that CKD changes therapeutic approach Recognition that bisphosphonates are not recommended in CKD Not recognizing that nephrology is already managing renal osteodystrophy
Bisphosphonate Holiday (occasionally denosumab as well)	“65 year old on alendronate for 14 years” “79 year old stopped IV zoledronic 2 years ago, BMD still low” “stopped risedronate 1 year ago, just fractured wrist” “77 year old BMD shows high risk but took alendronate for 15 years, 10 years ago. OK to re-start?” “60 year old with intermittent bisphosphonate use × 7 years, BMD says ‘high risk’” “72 year old on alendronate × 4 years but dentist says it must be stopped for 6 months to get implants but BMD T-score < -2.5” “how long is it safe to use denosumab?”	Duration of bisphosphonate therapy Monitoring of bisphosphonate holiday Duration of bisphosphonate holiday Response to monitoring change while on bisphosphonate holiday Fracture risk while on drug holiday Fracture occurrence while on drug holiday Medication re-start Over-use of bisphosphonates
Routine Osteoporosis Assessment	“52 year old seeking information about treatment options” “64 year old woman with BMD showing moderate risk” “patient is high risk but does not want to be treated; please see and advise” “patient requests referral for specialist opinion and education”	Uncertainty about intervention threshold Patient needs extra time/education Patient asking many questions Concern about non-treatment of low T-score BMD decreasing in postmenopausal woman Knowledge deficit for routine OP care Reassurance from specialist Making use of local expert resources/education Patient request to see specialist Doctor/patient disagreement on plan
Medication Options beyond oral bisphosphonate	“67 year old with gastrointestinal side effects from alendronate” “83 year old with muscle twitching after each risedronate dose” “55 year old with cirrhosis and varices, can’t risk oral bisphosphonate” “60 year old who refuses bisphosphonate because she already has jaw pain”	Typical adverse effects Atypical adverse effects True contradindications Educational deficit around potential risks for adverse effects
Adverse DXA change on therapy	“decrease in BMD despite alendronate” “2 years on IV zoledronic acide and BMD still shows osteoporosis” “BMD not getting better on therapy” “on therapy 4 years and BMD still shows ‘high risk’”	Knowledge deficit on clinical interpretation of small decreases in BMD Uncertainty about role of serial BMD testing Uncertainty about role of re-calculation of risk scores while on therapy Radiology narrative reports about failing therapy
Metabolic bone diseases	“low bone density in 29 year old man” “family history of metabolic bone disease” “Rheumatoid arthritis on long-term prednisone”	Osteogenesis imperfecta, Paget disease, parathyroid disorders, phosphate disorders, osteopetrosis Drug-induced – glucocorticoids, heparin, anti-epileptics Complex chronic disease – iron storage disorders, short gut syndrome, gastric bypass, transplantation, alcoholic bone, chronic liver disease Work-up for unexpected low BMD in young person Idiopathic male osteoporosis
Request for assessment and access to IV zoledronic	“patient interested in IV bisphosphonate”	Comparative efficacy of oral vs IV bisphosphonates Practical access to outpatient IV therapy
Specific request for anabolic therapy	“3 vertebral fractures, please assess for teriparatide treatment”	Access to drugs rarely used in primary care
Premature low estrogen state	“30 year old woman with premature menopause” “22 year old with severe endometriosis, requires ovarian suppression”	Role of BMD testing Options for bone mass maintenance outside of menopause
Serious adverse effect		Suspected osteonecrosis of jaw Completed or impending atypical femur fracture
Malnutrition related osteoporosis	“26 year old with severe anorexia nervosa and hip fracture”	No guidelines for management
Cancer therapy effect on bone	“54 year old woman with breast cancer starting aromatase inhibitor” “79 year old man with prostate cancer taking GnRH agonist” “32 year old with vertebral fracture 1 year post stem cell transplant”	Uncertainty from either primary care or oncology about bone management with cancer care
Immobilization	“38 year old with quadriplegia and 2 lower limb fractures in past year”	Not addressed in guidelines
Pain management	“77 year old with 2 vertebral fractures, please assist with pain management”	Limited access to acute pain management services

## Results

**Fig. 1:**
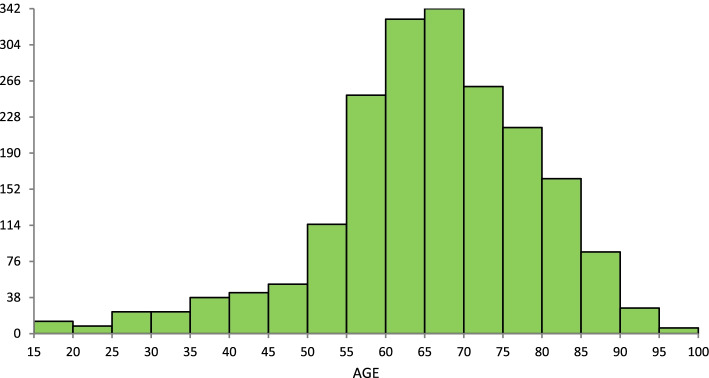
Frequency distribution of patients by age referred to the Osteoporosis clinic, 2015–2019

**Fig. 2:**
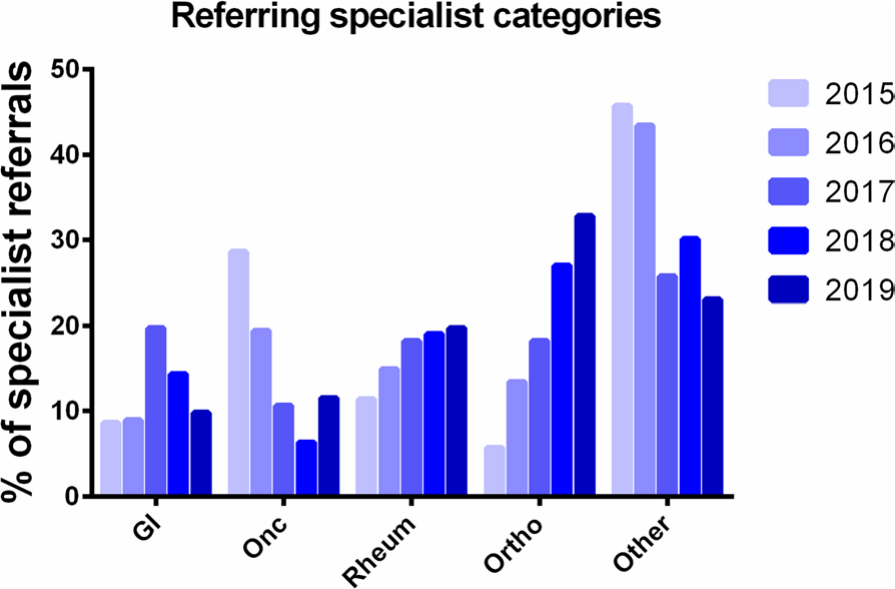
Specialty of non-primary care based referrals to the Osteoporosis clinic, 2015–2019. Note that “orthopedic surgery” includes referrals from the Fracture Liaison Service which started in 2017. GI, gastroenterology, Onc, oncology, Rheum, rheumatology, Ortho, orthopedic surgery. “Other” includes all medical or surgical subspecialists not otherwise specifically listed

During the 5-year sampling frame, there were an average of 1189 referrals received each year (total 5945). The typical patient demographic is displayed in Fig. 1 and Supplemental Table 1; there was no apparent change across 5 years. Approximately 87% of referred patients were women with a median age of 66 (range of 15 to 99) years. Note that 10% were patients under the age of 50 and most such referrals were for metabolic bone diseases (69.5%). Referrals from urban family practitioners comprised the majority (71%) with 13.7% from rural practitioners and 14.9% from other specialists. The most common referring specialists were gastroenterologists, oncologists, rheumatologists and the region-wide Fracture Liaison Service (FLS) that started in 2017 (Fig. 2), who refer on behalf of the orthopedic surgeons. Prior to the FLS program, orthopedic surgery accounted for 1.4% of referrals to the centre but by 2019, it was 5.0% (*p* = 0.0002).

**Fig. 3:**
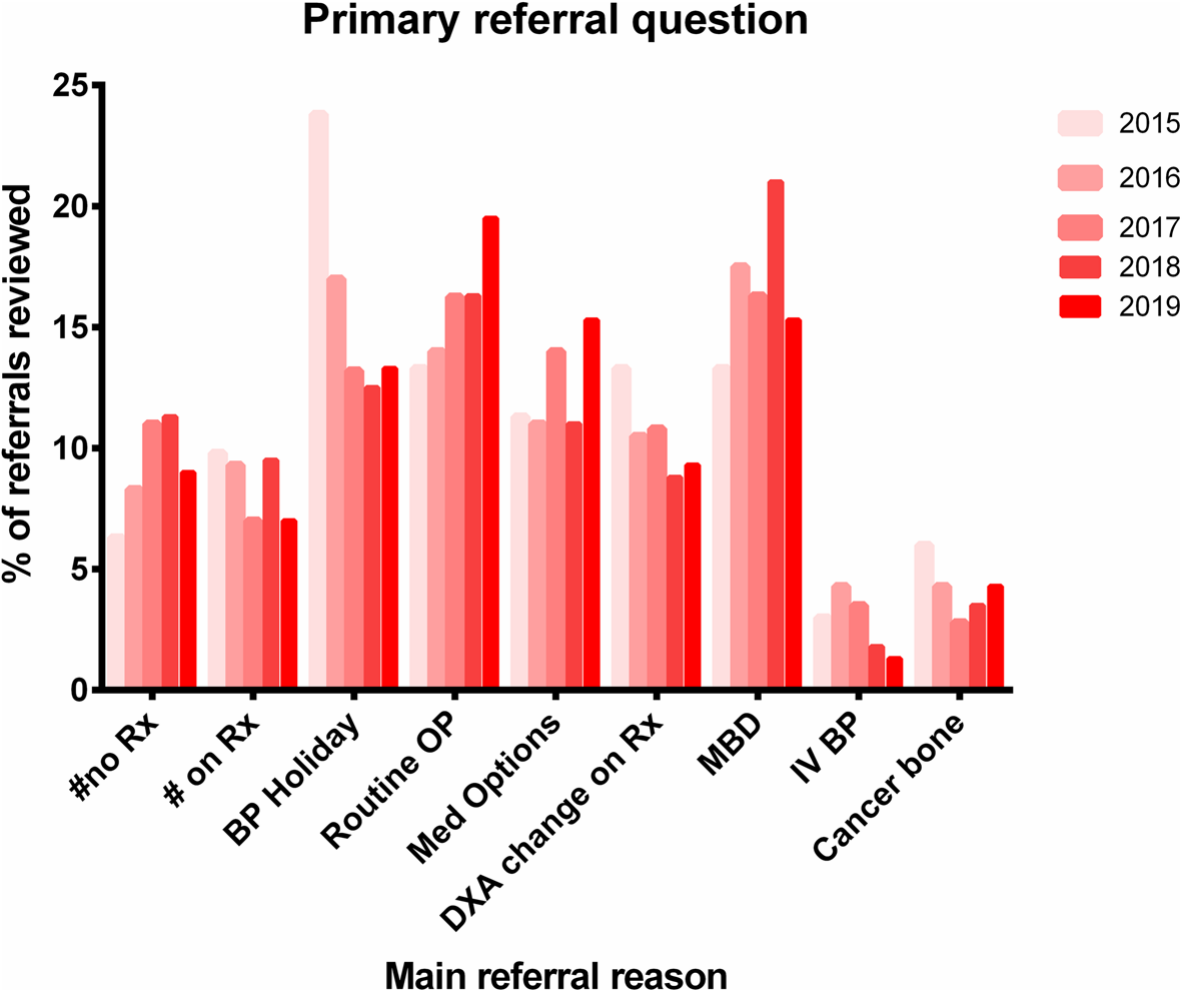
The nine most common primary referral questions, 2015–2019, by percentage of all referrals. See Table 1 for explanations and examples. #no Rx, fracture not on treatment, BP, bisphosphonate, OP, osteoporosis, DXA, dual x-ray absorptiometry, MBD, metabolic bone diseases, IV, intravenous

**Fig. 4:**
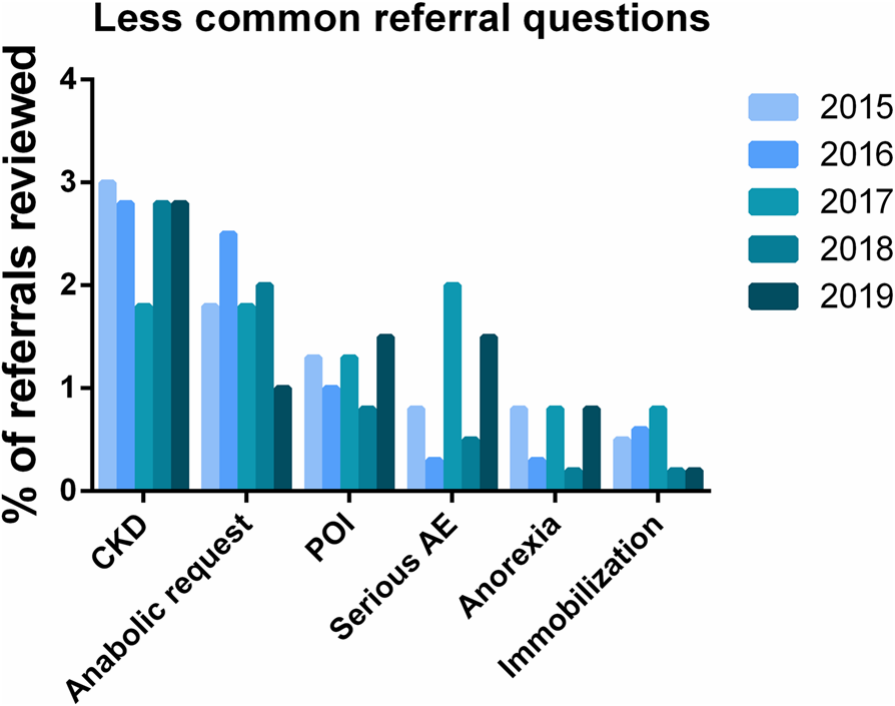
Less common primary referral questions, 2015–2019. See Table 1 for explanations and examples. CKD, chronic kidney disease, POI, premature ovarian insufficiency, AE, adverse event (atypical femur fracture, osteonecrosis of the jaw). Note that the 16^th^ category, pain management only accounted for < 6 referrals and is not shown)

**Fig. 5:**
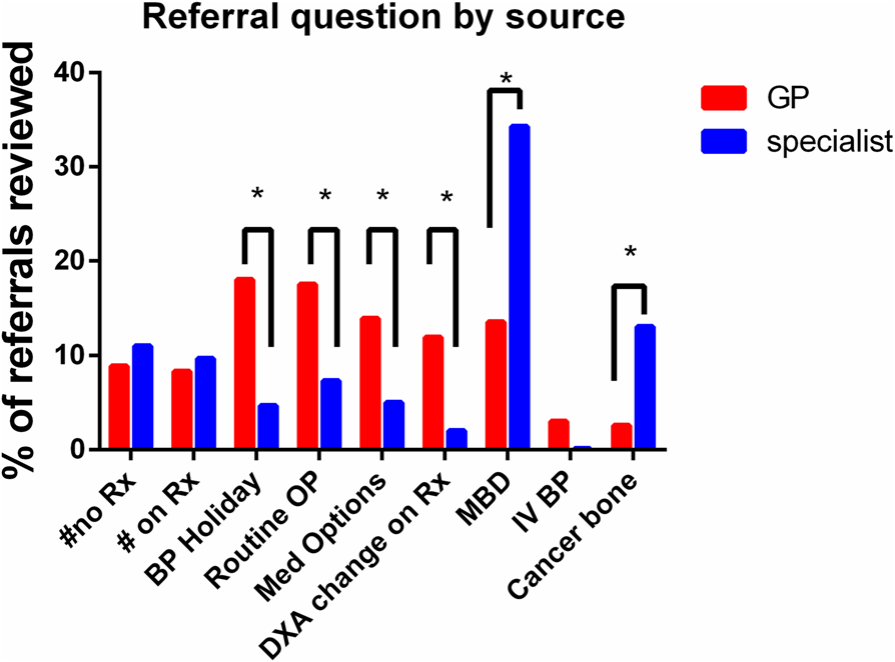
Most common referral questions according to whether the referral source was primary care or specialist. “*” indicates *p* < 0.05 by Chi-square test

The distribution of common primary referral questions is shown in Fig. 3 with less common primary questions in Fig. 4. This demonstrates that there are 7 question categories that account for 89% of all consultation requests to specialist bone medicine: 1) metabolic bone diseases [a composite category] (16.7%), 2) bisphosphonate holiday (15.9%), 3) routine osteoporosis assessment (15.9%), 4) non-bisphosphonate medication options (12.5%), 5) adverse changes on DXA despite therapy (10.5%), 6) recent fracture without treatment (9.2%) and 7) fracture while on therapy (8.5%). From 2015 to 2019, there appeared to be a decrease in the proportion of referrals for bisphosphonate holiday (24% to 13%, *p* < 0.0001) and increase in the frequency of requests for routine osteoporosis assessments (13% to 20%, *p* < 0.0001). Figure 5 demonstrates that there are significant differences in the services requested by family practitioners compared to specialists. Referrals from the community practitioners were more likely to involve requests for routine osteoporosis assessments or issues around bisphosphonate use whereas specialists accounted for the majority of metabolic bone disease and cancer-treatment-related bone disease (*p* < 0.001 for all comparisons).

Exploratory subgroup analysis of the age-distribution among different referral types showed that patients referred for “fracture on treatment” and “bisphosphonate holiday” were significantly older (median ages 73 [interquartile range 65–82] and 70 [64–77] respectively) than patients referred for “routine osteoporosis assessment” and “fracture without treatment” (median ages 63 [58–69] and 68 [60–77], adjusted *p* < 0.0001 for all comparisons).

## Discussion

We have performed a large-scale, multi-year audit of our regional bone medicine specialty clinic (the only such clinic in Southern Alberta) and elucidated a number of key issues in osteoporosis medicine that generate consultation requests. The osteoporosis clinic provided service to both primary care and a wide range of specialists, including support to hospital-based FLS programs, although there were differences in the types of patients referred from each source. Although this area of medicine is often considered as “osteoporosis and senior’s health,” a meaningful proportion of consultation requests arise for patients under age 50 and those with non-osteoporosis metabolic bone disease.

A review of primary referral questions helps osteoporosis specialists better understand the needs of their referring community and may give insight as to the most common areas in routine osteoporosis care where family practitioners may feel as though more guidance is required. It is remarkable that 6 of the top 7 primary referral questions pertain to issues expected to arise in routine osteoporosis care, such as what to do with the patient who sustains a fracture despite taking an approved anti-fracture medication. Fracture despite therapy is common and reported in every single randomized controlled trial [7] of anti-fracture agents. Even the best available therapy, which reduces relative fracture risk by 50–70% [8, 9], will still see some patients fracture, especially those who are elderly and at high risk for fracture [10].

Our subgroup analysis looking at age distribution around certain referral questions showed that fracture on therapy and issues of bisphosphonate holiday were particularly seen in an older population; this is not surprising and points out that simply by virtue of increasing age and duration of therapy, fractures and questions of drug holiday will be expected in many patients. Unfortunately, although they increasingly acknowledge the need for long-term patient management plans, most guidelines do not give specific recommendations that can address the many clinical permutations around bisphosphonate holiday, fracture on therapy or sequential therapy [3, 4]. The science regarding interpretation and use of serial BMD is very unsettled [11–14] and even high profile guidelines offer frankly contradictory advice on this issue [5, 15]. This has led some writers to call for more comprehensively useful guideline recommendations, even if such recommendations cannot be fully supported by the highest level evidence [16]. Better still, this kind of real-world data may be useful to help identify research questions that are particularly relevant and desired by actual patients with osteoporosis. Until such evidence exists, primary care physicians my need to rely on specialty clinic support. Given our findings, one may hypothesize that lack of accessible bone health education and osteoporosis clinical support may be a contributor to both poor osteoporosis therapy uptake and adherence to therapy by patients [17, 18]. Addressing this problem will require either increasing access to osteoporosis specialty care or improving the confidence for broader osteoporosis management within primary care. In either case, this will necessitate key stakeholders and policy-makers to support the development and implementation of widespread knowledge translation and education initiatives within the osteoporosis field.

In addition, our systematic analysis provides the infrastructure for the development of a service-driven teaching curriculum for training programs in osteoporosis and other metabolic bone disorders. In order to serve the needs of the medical community, it is clear that training around osteoporosis guidelines is not sufficient. Our data shows that the bone specialist needs to be familiar with controversies in the field, the practical use of the entire osteoporosis therapeutic armamentarium, the proper radiologic and clinical interpretation of DXA-BMD, complex secondary osteoporosis assessments in addition to a very long list of uncommon bone diseases which actually comprise a significant cumulative portion of the clinical needs.

Previous approaches to creation of a musculo-skeletal teaching curriculum have involved surveying practicing members within a specialty (e.g. orthopedic surgery) in order to determine what is deemed important for their own residents [19, 20] however, this only incorporates the specialists’ view and does not consider the actual needs of the referral community. Community-based studies involving mailed questionnaires [21, 22] structured interviews [23] and educational packages [24] have attempted to measure the osteoporosis “knowledge needs” in patients, primary care and specialists. However, all of these study designs are subject to recall bias or the fact that many providers may not know what they do not know. Our approach to defining knowledge needs according to an actual review of real-patient referral questions is a novel approach to comprehensively defining the breadth and frequency of osteoporosis medicine topics in real-world practice. As osteoporosis potentially affects 77% of people over the age of 80 [2], the need for osteoporosis care is similar to what is required for other diseases of ageing such as cardiovascular disease or cancer therapies [25].

The greatest strength of our study is the large size; 2000 referrals reviewed gives sufficient ability to derive reasonable prevalence estimates of even uncommon referral questions- this is particularly useful to contextualize our study as an exercise in osteoporosis curriculum design. There are also some limitations to be considered; it is possible that five years may be too short an interval to observe long-term trends in referral questions arising in parallel with new directions in the osteoporosis field. Anecdotally, bisphosphonate holiday questions were hardly seen fifteen years ago. The referrals in this study were interpreted by a single experienced reviewer which aids with consistency of approach but could also introduce systematic interpretation bias. Nonetheless, even multiple reviewers could have mis-categorized referrals when the data or narrative is unclear or sparse in detail and referral quality was not defined or used as an inclusion criteria in order to avoid bias in selection for review. Some referrals could theoretically qualify in 2 or 3 question categories once assessed in person and it is impossible to know if the referral source intended or was aware of this; it is possible that the referral was sent due to multi-aspect complexity even if not explicitly stated. However, if anything, this emphasizes the reality of patient care in osteoporosis – patients’ problems might not be amenable to uni-dimensional categorization and osteoporosis guidelines and training will need to reflect a more complex integration of osteoporosis medicine principles in order to be truly useful for real-world patient care.

## Summary

We conducted a large review of patient referrals sent to a full-service metabolic bone clinic over 5 years and found there are 7 common sub-groups of primary referral questions pertaining to osteoporosis care but also complex metabolic bone diseases. This real-world data provides important information to inform guideline writers, knowledge translation groups and specialty curriculum designers in osteoporosis care. Community-oriented training in osteoporosis is recommended in order to provide optimal support for the care of medical bone disorders in Canada. Primary care providers shoulder the majority of osteoporosis care in Canada and should be offered attractive and clinically useful tools to guide their decision-making across the entire span of osteoporosis care from screening to therapy end.

## Supplementary Information

**Additional file 1: Table 1.** Basic demographics of patients referred to the Osteoporosis clinic.

## Data Availability

Not publicly applicable to the present manuscript as it is contained within unique patient referral letters sent to a clinical osteoporosis centre. All data generated or analysed during this study are included in this published article.
