# Supplementary material for: A retrospective review of the community medicine needs from osteoporosis services in Canada
Source: BMC Endocr Disord. 2022 Mar 26;22:78. doi: 10.1186/s12902-022-01000-y (PMC8961948; doi:10.1186/s12902-022-01000-y)
Supplement: Supplementary file 1 — Additional file 1: Table 1. Basic demographics of patients referred to the Osteoporosis clinic. [file 12902_2022_1000_MOESM1_ESM.docx]

Supplemental Table 1: Basic demographics of patients referred to the Osteoporosis clinic.

| YEAR | Median age | Range, age | Female (%) |
| --- | --- | --- | --- |
| 2015 | 65 | 18-99 | 87% |
| 2016 | 67 | 18-97 | 85% |
| 2017 | 66 | 15-96 | 86% |
| 2018 | 65 | 18-96 | 89% |
| 2019 | 66 | 18-92 | 89% |
